# Gene × Environment Interaction in Developmental Disorders: Where Do We Stand and What’s Next?

**DOI:** 10.3389/fpsyg.2018.02036

**Published:** 2018-10-26

**Authors:** Gianluca Esposito, Atiqah Azhari, Jessica L. Borelli

**Affiliations:** ^1^Psychology Program, Nanyang Technological University, Singapore, Singapore; ^2^Department of Psychology and Cognitive Science, University of Trento, Trento, Italy; ^3^Department of Psychological Science, University of California, Irvine, Irvine, CA, United States

**Keywords:** gene × environment interaction, developmental disabilities, environmental hazards, genetic risk, self-generated environment, developmental trajectories, treatment outcomes

## Abstract

Although the field of psychiatry has witnessed the proliferation of studies on Gene × Environment (G×E) interactions, still limited is the knowledge we possess of G×E interactions regarding developmental disorders. In this perspective paper, we discuss why G×E interaction studies are needed to broaden our knowledge of developmental disorders. We also discuss the different roles of hazardous versus self-generated environmental factors and how these types of factors may differentially engage with an individual’s genetic background in predicting a resulting phenotype. Then, we present examplar studies that highlight the role of G×E in predicting atypical developmental trajectories as well as provide insight regarding treatment outcomes. Supported by these examples, we explore the need to move beyond merely examining statistical interactions between genes and the environment, and the motivation to investigate specific genetic susceptibility and environmental contexts that drive developmental disorders. We propose that further parsing of genetic and environmental components is required to fully understand the unique contribution of each factor to the etiology of developmental disorders. Finally, with a greater appreciation of the complexities of G×E interaction, this discussion will converge upon the potential implications for clinical and translational research.

## Introduction

Neurodevelopmental disorders emerge from numerous genetic and environmental sources, which begin to exert their effects at the embryological and early fetal stages of life (e.g., Brimacombe et al., 2007; Gardener et al., 2009; Esposito and Borelli, 2018). In the clinical setting, deciphering precise etiological pathways is not currently possible. Within the research context, newer screening technologies afford a continual shift from simplistic conventional nature-versus-nurture perspectives toward a nuanced framework of gene-by-environment interactions (G×E) – this shift promotes a more accurate understanding of the complexity of etiological pathways.

To illustrate the genetic complexity mired in the etiology of psychopathology, we must first develop an appreciation of the numerously intricate mechanisms of gene expression. At the level of the genetic structure, mutations in the DNA, such as a change in a single nucleotide base, may cause a significant alteration in the three-dimensional molecular structure of the expressed protein, potentially leading to drastic changes in the organism’s traits. On other occasions, the extent to which such mutations affect the individual is dependent upon existing compensatory mechanisms of other interacting genes and gene products (i.e., RNA and proteins) within the same biological pathway, with similar overlapping functions and expression patterns (for a review, see Tautz, 1992). Furthermore, the nature of interaction between genes or gene products may differ according to the temporal (i.e., developmental period), biochemical (i.e., active or resting state of protein) and spatial (i.e., location of interaction within the body) parameters present during gene expression. Genes often do not possess a one-to-one relation with the characteristics that they contribute to (Morange, 2007), and this is the prevalent case for complex behavioral traits, and especially so for developmental disabilities.

In the quest to elucidate specific mechanisms that underpin psychological disorders, scientists have ventured beyond the scope of genetics, and mounting consideration has been paid to the interaction of genes with hazardous environmental factors. Specifically, grasping the concept of adaptation and developmental plasticity necessitates a deliberation of the impacts of genetic determinants, whether alone or in combination with environmental risk factors (i.e., maternal illnesses, maternal nutritional status and environmental toxins). Over the long term, appreciation of specific G×E mechanisms underlying neurodevelopmental disorders should result in more effective risk-mitigating or preventive interventions (Graf et al., 2013), but this promise has yet to be realized, in part because of the complexity of the relations among these factors. On one hand, studies have demonstrated that most cognitive traits as well as psychiatric disorders are moderately to highly heritable (Bishop, 2009); nevertheless, efforts by researchers to identify genes that account for significant portions of variance in these disorders have been fraught with difficulties and have yet to come to fruition. On the other hand, according to the hypothesis of differential susceptibility, the environment is full of potentially hazardous factors, but these factors only affect people with specific genetic predispositions that render them more susceptible to harmful environmental influences (Belsky and Pluess, 2009). Furthermore, many psychiatric disorders are neurodevelopmental in their origins: Human and animal studies point to the importance of a critical period during which neural circuits (that develop from genetic information) can be potentially affected by environmental hazards. In other words, there is a highly plastic critical period, which is a time of great responsiveness of the central nervous system to adverse events (Leonardo et al., 2007; Esposito et al., 2017b; Truzzi et al., 2017, 2018). Similarly, the expression of genetic factors may exert a specific effect in a time-dependent manner. An example of a neural structure that matures in the immediate postnatal period of infancy is the superior olivary complex (SOC), which is responsible for the onset of spatial hearing. Located in the brainstem, the development of this structure occurs over a critical time-window, during which extensive upregulation in the gene expression of serotonin-related genes and genes associated with the peripheral auditory system accompany this maturation (Friauf, 2004). Infants with an underdeveloped SOC may not be able to localize auditory cues from their surroundings, which in turn predispose them to auditory processing disorders and developmental disabilities, including dyslexia and autism spectrum disorder (ASD) (Tallal, 2012). Illustrations such as these lead us to consider G×E as a fluid model where the temporal dynamics play a very important role.

The great challenge in this field is related to how to conduct assessments of G×E relations, and to identify and subsequently set ideal standards for collecting, analyzing, and interpreting the data. When considering G×E interactions in research studies, the very first consideration is how to select the candidate gene/s to study. One pre-requisite of candidate gene selection is the availability of gene-disease databases from an array of past epidemiological and animal model research, as well as linkage and gene expression studies (Green and Moore, 2006). While web resources may expediate the selection of candidate genes, a robust understanding of genetics and neuropsychiatric pathogenesis is nonetheless necessary for optimal selection (Green and Moore, 2006). Which leads us to ask the question, *what can psychology and psychiatry contribute to the study of G×E interactions of developmental disorders?*

The goal we pursue in this perspective paper is to provide a framework that scientists can employ to design feasible theoretically driven studies assessing G×E interactions that have the potential to contribute to the field of developmental psychopathology. It is worth noting that in this paper, we refer to psychopathology as defined by DSM-5 criteria, but we believe that our recommendations are equally relevant for researchers interested in pursuing Research Domain Criteria (R-DoC) – informed projects pertaining to psychological dysfunction. Initiated by the National Institute of Mental Health^1^, R-DoC is an unprecedented methodological approach which associates psychiatric problems with symptoms (e.g., low social functioning) instead of psychiatric categories (e.g., depression, borderline personality disorder) (Cuthbert, 2014, 2015). In theory, R-DoC should allow investigation into the causes of psychiatric problems to be liberated of the inaccuracies of diagnostic categorization. In this manner, R-DoC enables research into the basic mechanisms of psychiatric disorders, transcending traditional psychiatric classification (Insel and Cuthbert, 2009; Kozak and Cuthbert, 2016). Recently, studies on developmental disabilities, such as autism spectrum disorders (ASD), have begun to incorporate the R-DoC paradigm (e.g., Montalvo-Ortiz et al., 2015; Foss-Feig et al., 2016). However, to maintain consistency with the majority of the studies we cite in this review, we describe pathology in terms of psychiatric disorders. With the availability of more developed technological and statistical tools, researchers can move beyond employing singular methods examining psychological disorders, and can utilize our suggestions to inform their pursuit of G×E research. In the sections that follow, we provide our recommendations for such a research agenda.

## Terminology in G×E

To facilitate comprehension, three key terms will be introduced in this section: single-nucleotide polymorphisms, heritability and phenotypic variance. Firstly, single nucleotide polymorphism (SNP) is a DNA polymorphism that is most typically explored in numerous G×E studies. SNP is a variant in a DNA sequence that occurs commonly in the population (e.g., 1%) in which a single nucleotide base differs between members of the population (e.g., A versus G). Secondly, the term *heritability*, an important concept in G×E studies, refers to the extent to which genetic differences passed down from relatives, as compared to environmental factors, contribute to observed phenotypic differences between two or more individuals. Variations in a certain quality (e.g., height) within the population that are not accounted for by genetic factors could be due to environmental variables, a combination of both factors (such as in G×E interactions), or non-genetic contributions (i.e., residual effects). Lastly, a closely linked concept that is intuitive to the idea of G×E interaction is phenotypic variance. Phenotypic variance typically accounts for the combinatorial effects of genetic and environmental variance in explaining a specific presenting trait.

## Theoretical Frameworks: Diathesis-Stress Vs. Differential Susceptibility

Theoretical underpinnings within the G×E field usually fall into one of the two most prominent categories: the diathesis-stress or the differential-susceptibility model. The diathesis-stress model stipulates that an individual who possesses genetic vulnerability may be susceptible to a psychological disorder when exposed to an adverse environment, but the disorder does not manifest without the trigger of an environmental stressor. However, individuals without predisposing genetic susceptibility do not develop a psychological disorder even when faced with adverse environmental conditions. While this model has stimulated exciting research in this field, its disproportionate focus on negative life events, disregarding positive environments, render it myopic in its scope (Belsky and Pluess, 2009). An alternative theoretical framework, the differential-susceptibility perspective, was subsequently proposed (Belsky et al., 2007; Belsky and Pluess, 2009). Instead of suggesting that certain genotypes are intrinsically good or bad, this model proposes that individuals’ susceptibility to environmental effects (both negative and positive) differ depending upon genes that are involved in responsivity to environmental states, coined as “plasticity genes.” Specifically, plasticity genes can either aggravate the risk of psychopathology in negative environments, or alleviate the risk of psychopathology in positive environments, such that the most distressed individual in an undesirable environment is also the one who is most likely to be aided in a positive environment (Belsky and Pluess, 2009).

## Challenge One: Selecting Genetic Variant(S)

Perhaps the first step in beginning the process of designing a G×E study is to decide what gene(s) to study. This decision is greatly influenced by the method employed in conducting the G×E study, in which there are commonly two approaches from which to choose: candidate-gene studies or genome-wide association studies (GWAS). Candidate-gene studies typically focus on individual SNPs based on the argument that these variants have a high likelihood of being implicated in the biological mechanism through which the psychiatric disorder manifests. Some examples of established methods that have been adopted to investigate SNPs in G×E studies include the bivariate linear mixed model (Lee et al., 2015, 2017) and the random regression linear mixed model (Robinson et al., 2017). In the former, association analyses are conducted on SNPs and two environmental traits (e.g., winter-born, not winter-born) in cases and controls. The latter approach, by comparison, is more sophisticated and is based on an algorithm that allows for multivariate analysis of complex traits within the context of multi-trait models (Lee and van der Werf, 2016).

However, each genetic variant usually accounts for only a small slice of the genetic variation of the disorder (Duncan and Keller, 2011). Candidate-gene studies are usually conducted with small sample sizes and thus possess smaller statistical power as compared to GWAS studies. A large proportion of the existing G×E literature comprises of candidate-gene studies, possibly reflecting an increased interest in elucidating specific etiological pathways underlying psychiatric disorders with respect to the genetic polymorphisms under investigation. On the other hand, GWAS studies are conducted with large sample sizes (>1,000) and boast significantly higher statistical power, with no prior conception of specific genetic variants to target. Indeed, GWAS analyses usually scan entire genomes consisting of thousands of individuals to identify numerous associations of genetic variants with specific behavioral traits.

The past decade has witnessed a promising increase in the number of whole-genome screenings being conducted, set within the framework of G×E interactions (Aschard et al., 2012). To estimate the percentage of phenotypic variance from such GWAS studies, a conventional method compares the coefficients of determination across statistical models that either omit or include significantly related variants. The challenge in this approach is that it requires extensive genotypic and phenotypic data which is difficult to procure. Indeed, while genome-wide investigations of developmental disabilities represent the most complex and thorough method of understanding multiple etiological factors underlying developmental disabilities (Munafò and Flint, 2010; Cross-Disorder Group of the Psychiatric Genomics Consortium, 2013; Flint and Munafò, 2013), such designs necessitate larger samples than are typically enrolled in psychological studies.

These types of research projects may be possible if psychologists can collaborate across sites, first conducting genome-wide analyses of smaller samples (e.g., *N* = 100), before pooling these samples together for the purpose of genetic analyses. To facilitate this type of work, we have created a listserv for researchers interested in G×E research in developmental psychopathology (to subscribe, contact the first author). On this listserv, researchers can find colleagues with similar interests in collecting genetic data from participants in cross-sectional or longitudinal studies and can coordinate across studies (in terms of measure/outcome selection, method of collection of genetic material, etc.). Researchers can also forge collaborations with geneticists and assist them in using gold-standard psychiatric measures to assess children’s psychopathology in their studies. We hope that this platform will facilitate greater collaborative work which will accelerate progress in this field.

In recent years, an alternative approach to genome-wide analyses has surfaced, utilizing only the summary statistics of GWAS G×E studies. In 2016, two groups of researchers, Pare et al. (2016) and Shi et al. (2016), formulated statistical methods to estimate marginal genetic variance, while simultaneously accounting for sample size limitations, linkage disequilibrium between variants of interest and correlation matrices of SNPs. This promising approach has been further extended by Laville et al. (2018), who implemented statistical packages that aid in the estimation of proportion accounted for by G×E interactions from GWAS summary statistics. In the absence of the ability to undertake a genome-wide analysis, it may be prudent to first understand which genes are most likely to have associations with psychopathology. A good place to start this investigation is with the genetic material that has been described as “generalist genes” (Kovas and Plomin, 2007). Generalist genes are a collection of genes which greatly account for genetic influence on a number of developmental disabilities (e.g., FOXP2, COMT, GABRB3, SHANK1-3, and DISC1). Studies have shown that alterations to this same group of genes exerts a fundamental genetic influence on a number of developmental disabilities, ranging from ASD to schizophrenia (Wang et al., 2016), to intellectual disability (Bhowmik et al., 2011) and attention deficit and hyperactivity disorder (ADHD) (Brinksma et al., 2017; Heinrich et al., 2017). However, the same alterations may also be found in the typical population – in other words, the presence of these genetic alterations alone is not sufficient to predict the resulting pathological phenotype. Indeed, what seems to precipitate the emergence of a typical or atypical phenotype is largely accounted for by the interaction of the environment with these genetic anomalies (Caspi et al., 2002, 2003). Broadly, environmental factors can be categorized into those that represent unwanted external hazards and those that are generated by the individuals (e.g., substance abuse). With this degree of environmental variables, investigators should work towards greater conceptual clarity under this dichotomous framework – either by more concise operationalization of terms or clearer categorization.

Having previously illustrated the complexity of genetic expression in manifesting a phenotype, whereby genes rarely have a one-to-one relation with a specific trait, a more holistic experimental design involves a polygenic approach. Polygenic inheritance refers to features and traits of an individual that develop due to the cumulative effect of several genes. Polygenic traits, such as skin color and behavioral characteristics, differ between individuals by such slight gradations that they are considered to be continuous in nature. While investigation of hypothesis-driven candidate genes has been recommended (Rutter et al., 2006), there is great likelihood that multiple genes function together to mold the genetic risk of developing a psychiatric disorder (Kraft and Aschard, 2015). This approach offers an exciting alternative to designing multigenic G×E studies as it simultaneously considers the contribution of multiple genetic variants commonly linked to a specific psychiatric disorder. Operationally, polygenic analyses are accomplished by tabulating the number of alleles for each SNP and weighting the sum by the effect size obtained from a GWAS, to generate a polygenic risk score (PRS). A higher PRS value indicates a greater genetic predisposition toward developing a psychiatric disorder. Thus, the PRS essentially is a numeric representation of the additive effect of several SNPs, reflecting an individual’s genetic profile risk more accurately as compared to single candidate gene analyses. Recently, studies that employ a polygenic approach have elicited larger effect sizes and predictive power (Bulik-Sullivan et al., 2015; Maier et al., 2015). Alas, while the polygenic approach allows for a more complete impression of the genes it does so at the expense of finer genetic resolution, as it leaves investigators unable to identify specific genes associated with the disorder. However, complementary techniques at the level of singular genes can be conducted to supplement polygenic approaches, revealing the unique contribution of relevant variants.

## Challenge Two: Selecting the Design

The next step in designing G×E research involves identifying the ideal methodology for examining research questions. We discuss several types of research designs for assessing G×E interactions. The first type of design we consider here is case–control studies. Case–control studies employ correlational analysis to assess the difference in outcomes across two groups. The groups are determined based on genotype – participants who differ in one characteristic (e.g., a specific genotype) are distributed across groups. Investigators then compare participants in these two groups to determine whether this sole differentiating feature (group membership) is responsible for a significant percentage of the variance in the differences between these groups. Although gene–environment interactions may be investigated using case–control paradigms, an issue of concern is choosing of appropriate control subjects for these studies. In case-control studies, the control group (the group lacking the feature under consideration) needs to meet specific and rigid criteria to ensure that eventual differences in the outcomes are actually due to the investigated feature rather than to other spurious variables (for example, participants’ age, cultural group, educational level, socio-economic status, or people’s past, and current health). Therefore, a thorough consideration of control group characteristics should be performed and inclusion criteria should be tailored to each study to account for the specific characteristics of the considered population. To illustrate the complexity of identifying a case control group, consider the difficulty of finding a control group for a sample of depressed children – should this control group exclude children with anxiety-related symptoms or pathology given the extremely high rates of comorbidity between depression and anxiety (Unick et al., 2009)? Doing so will undoubtedly reduce the generalizability of the findings, yet the creation of four groups (depression only, depression plus anxiety, anxiety only, and no depression or anxiety) necessitates a significantly larger sample. Further, even if this fine-grained clinical distinction is made, there are undoubtedly numerous other variables on which these two groups differ that could be confounded with the variable of interest.

There are also non-traditional study designs, such as those that do not include a control group, which can prove useful to investigators who are interested in adopting a dimensional approach. As compared to a categorical paradigm that presumes distinctive typical and abnormal mental health states, a dimensional approach assumes that traits lie on a continuous spectrum, and one is only diagnosed with a disorder upon presenting symptoms that fall beyond the normative threshold. A dimensional perspective is consistent with the prevailing view of developmental psychopathology. Researchers working within this approach can utilize the following study designs: (a) the case-only study, (b) the case-parental control study, and (c) the affected relative-pair study. Case-only studies allow for the investigation of the association between a genotype and exposure to an environmental variable among case subjects only. For instance, this could involve recruiting a sample of youth with social anxiety disorder and examining the presence of the oxytocin receptor gene (OXTR) in combination with parental overcontrol, an environmental risk factor involved in the emergence of anxiety in children (Borelli et al., 2015). To calculate additive effects of psychopathological risk, due to simultaneous incidence of adverse environmental and genetic factors, odds ratios are interpreted as a synergy index, with the environment and the genotype assumed to be independent of each other (Rothman, 1986).

In the absence of a direct association between genotype and disease, case-parental control studies allow for the comparison of genotypic distribution of case subjects with the expected distribution based on parental genotypes. For instance, researchers may wish to examine the children of parents with and without autoimmune diseases for their risk for ASD (for a review, see Chen et al., 2016); information on case subjects’ exposure status may be utilized to stratify the effect of a genotype.

In affected relative-pair studies, such as in twin-studies, comparison between allelic distribution, identical by descent between pairs of affected relatives, is made in contrast with the expected distribution in the case of an absence of genetic linkage between the locus and the disease; comparative analysis can be stratified according to extent of environmental exposure (i.e., exposure status). Twin-studies allow researchers to estimate the contributions and additive effects of genetic and environmental factors upon the emergence of the stipulated phenotype. An example of this can be observed in the study by Ivanov et al. (2017), where a twin-studies design was utilized to assess the influence of gene and environment on the emergence of hoarding symptoms.

Most of the methods above possess certain limitations, such as linkage disequilibrium (Slatkin, 2008), exceptions to the assumption of simple Mendelian transmission (e.g., polygenic traits), an inability to measure exposure effects accurately (Wong et al., 2004; Lobach et al., 2008; Holmans et al., 2009), the lack of availability of high-throughput environmental data (Patel et al., 2013) and imprecision of the type of G×E interaction being investigated: multiplicative scale (i.e., ratio measures of association) as compared to an additive scale (i.e., absolute difference measure of association). Despite these shortcomings, they serve as important tools to assess G×E etiology in disease states (Khoury and Flanders, 1996).

In the past decade, the advent of GWAS, which analyzed different individuals’ genetic variants in order to associate a variant to a particular trait, has also driven the evolution of G×E research. Although GWAS did not characterize environmental factors, studies on G×E interactions nonetheless benefitted from drawing upon the vast body of data generated from GWAS. These studies have begun to complement findings from GWAS by integrating environmental data, including a substantial focus on exposure assessment, with relevant genetic variants that have been discovered (e.g., Thomas, 2010). Genetic variants commonly investigated in G×E research are SNPs.

However, as previously mentioned, etiological analyses of disorders based on a single genetic and environmental contributing factor represents a hasty oversimplification of G×E interactions. Recently, multigenic studies, which employ a polygenic approach that aggregate genetic markers across an array of SNPs, have emerged to reveal G×E signals which have previously gone undetected in individual SNP analyses. For instance, Guo et al. (2017) utilized PRSs to examine a combined GWAS study on common genetic markers underlying ASD and obsessive–compulsive disorder (OCD). Additionally, greater emphasis on characterizing environmental factors has led to more comprehensive G×E studies that employed a Multi-G–Multi-E framework (Dai et al., 2013; Naj et al., 2013; Patel et al., 2013; Velez Edwards et al., 2013). The continual progress of the field in recent years has also prompted the development of more advanced G×E statistical models using GWAS data, such as the mixed model for polygenic interactions (G×EMM) that aggregates minor G×E interactions distributed across various parts of the genome, thus possibly capturing “missing” heritability (Dahl et al., 2018). Another statistical model, which has emerged from the genomics era, is the structured linear mixed model (StructLMM), which efficiently computes the complex interaction of genetic loci with multiple different environments (Moore et al., 2018). With a growing interest in the G×E etiology of psychiatric disorders, a proposed model to address this need is the multivariate reaction norm model (RNM). The RNM allows for the joint modeling of genotype and covariate (i.e., environmental risk factor that affects complex trait). As the covariate is also modulated by genetic and environmental factors, a joint model approach allows for the simultaneous investigation of correlation and interaction effects (Ni et al., 2018).

The complexity of G×E findings in the field of developmental disabilities will be optimally captured from meta-analytic studies, which have the advantage of critically assessing the theoretical and statistical soundness of any given G×E study based on systematically pooled information. However, methods for the meta-analysis of studies investigating interactions are not well developed (Taylor and Kim-Cohen, 2007). In addition, procedures to determine the sample size needed to detect gene–environment interactions are still not well defined (Yang et al., 2003). We encourage researchers, particularly those without significant grant funding, to consider embracing meta-analysis as a design of choice – conducting a meta-analysis enables researchers to become acquainted with the field, to identify gaps in the literature, and to provide a useful empirical synthesis of the observed pattern of effects.

## Challenge Three: Selecting the Environmental Factor(s) of Interest

In addition to identifying gene(s) of interest and the research design, it is also important to devote significant thought to identifying the environmental factor(s) of interest and having a clear conceptualization of what that environment, and the resulting G×E interaction, would represent. In the event in which the causal direction of the environmental variable in question is not unequivocally known, investigators may opt to apply a Mendelian Randomization (MR) method to first determine the direction of effect prior to the G×E investigation (Davey Smith et al., 2005). This method capitalizes on common genetic polymorphisms that have been found to modulate patterns of exposures (e.g., tendency to consume alcohol), which allows us to establish an association between a particular genotype and an intermediate phenotype. Such information may provide a more robust theoretical ground upon which G×E analysis can then be conducted.

Moving beyond this basic nature-nurture question, multivariate genetic analyses have revealed that genes serve as ‘generalists’ that broadly determine the range of one’s learning capacity (especially in the domain of learning abilities and disabilities), while environments act as ‘specialists’ which fine-tune an individual’s eventual aptitude.

### Environmental Hazards

Environmental hazards are defined as substances or events which have the potential to threaten one’s environment and, in so doing, adversely affect health. Recently, several epidemiological studies have demonstrated that environmental factors during the fetal phase, through early childhood, may modulate the risk of developmental disorders as well as diseases that will onset in adulthood (Brimacombe et al., 2007; Gardener et al., 2009). Numerous researchers around the world are interested in elucidating the long-term outcome of interactions between genes and early exposure to environmental hazards. Amongst them, there has also been a renewed focus in investigating medical and psychiatric conditions (Moffitt et al., 2005; Rutter, 2010). For example, in a recently published article (Sakurai et al., 2016) of a large cohort in Japan (Chiba study of Mother and Children’s Health: C-MACH), the authors utilized multi-omics analysis, in which they evaluated multiple datasets from the genome, metabolome, DNA methylation in the umbilical cord (epigenome), gut microbiome and chemical (environmental) exposure. The authors sought to model the onset of a number of conditions, including obesity, allergies, metabolic, endocrine, and developmental disorders. Another example of a large-scale longitudinal study is the Twins’ Early Development Study (TEDS). Using both multivariate quantitative and molecular genetic perspectives, TEDS investigated behavior problems and delayed development of linguistic, cognitive and academic capacities within the range of normal variation (Oliver and Plomin, 2007). TEDS data indicated that genetic and environmental factors have pertinent bearing in almost all areas of behavioral development. Moving beyond this basic nature-nurture question, multivariate genetic analyses have revealed that, especially in the domain of learning abilities and disabilities, genes serve as ‘generalists’ that broadly determine the range of one’s learning capacity, while environments act as ‘specialists’ which fine-tune an individual’s eventual aptitude. Consequently, while the environment influences differences in performance within and between learning abilities and disabilities, genes greatly impact similarities in performance across age (Oliver and Plomin, 2007).

Environmental hazards can be categorized into four types: (i) chemical, (ii) physical, (iii) biological, and (iv) psychosocial. An example of a chemical environmental hazard is exposure to pesticides, whereas an example of a physical environmental hazard is the presence of a strong electromagnetic field. A biological environmental hazard is something that alters the biological environment, producing a negative effect on health conditions, an example of which would be the presence of a pathogen (i.e., ebola). Finally, an example of psychosocial environmental hazard is extreme poverty.

One advantage of examining environmental hazards in G×E studies is that it is less likely that genetic variables contribute to the environmental factor, suggesting greater independence of influence on outcomes. For instance, while prenatal infections (e.g., rubella, herpes simplex virus, cytomegalovirus, and toxoplasmosis) disrupt fetal neurodevelopment, forming a possible etiological mechanism for psychopathology such as mental retardation, learning disabilities, and schizophrenia (Klein et al., 2006), there is a lower probability that gestational exposure to these infections is associated with individual differences that have genetic roots. However, we argue that even with respect to hazardous environmental factors, it is still important to account for the potential influence of genetics on environmental self-selection. Generally, individuals with psychiatric disorders are at an increased risk of engaging in sexual risk behaviors (Shrier et al., 2012), making them more likely to acquire these infections (Cunningham et al., 2017). Indeed, Clarke et al. (2009) reported that, on its own, prenatal exposure to pyelonephritis, a urinary tract infection (UTI) that is usually the result of sexually transmitted diseases, did not lead to a significant increase in risk of schizophrenia. However, the risk of developing schizophrenia increased fivefold in infants from families with a history of psychosis and who were gestationally exposed to this infection. Therefore, it is possible that children who have greater exposure to these prenatal infections also have stronger genetic loading for psychiatric disorders, which could render the examination of gestational exposure to infections as a genetically influenced environmental factor. To explicate yet another layer of complexity, it is critical that we address the possibility for hazardous environmental factors (e.g., substance use, smoking or alcohol) to exert epigenetic effects (mechanisms that modulate level of gene expression without changing the genetic code) (Rosen et al., 2018). Indeed, several researchers have discovered that the profile of DNA methylation and mechanisms of histone modification differ between heavy consumers of alcohol and healthy persons (Zhang et al., 2013; Lohoff et al., 2017). The modulation of gene expression by environmental exposure presents another mechanism, G–E correlation, to the complex phenomenon of G×E interaction, that should be further parsed apart.

#### Exemplar One: Autism Spectrum Disorder

Despite the issues related to methodological limitations, studies have reported useful results that have increased our knowledge on the G×E interaction in the ontogeny of developmental disorders. Among the early environmental factors, maternal lifestyle and prenatal factors play important roles and may trigger serious health consequences and diseases later in life (Barua and Junaid, 2015). Some of the factors that have been found to influence normal fetal development include stress, diet, gestational diabetes, and exposure to alcohol during pregnancy (e.g., Barker, 2006; Bose et al., 2017; Salihu et al., 2017; Thompson et al., 2017). Unhealthy lifestyles generate epigenetic changes, including DNA methylation alteration and chromatin modifications, which are believed to account for various types of developmental disabilities related to brain plasticity, including neural tube defects and ASD (Dunaway et al., 2016; Cataldo et al., 2017, 2018).

Autism spectrum disorder was initially thought to be a result of environmental factors. However, genetic factors have been increasingly considered to play a more pivotal role in the etiology of autism, a discovery which is largely owed to recent discoveries of genetic mutations that implicate the encoding of synaptic molecules which relay communication between neurons. Recent studies have explored the role of epigenetics in the development of ASD (e.g., Dunaway et al., 2016). Epigenetics is a mechanism that influences gene expression without changing DNA nucleotide sequence, but by modifying the expression of the gene by non-genetic influences (Berger et al., 2009). As epigenetic changes are, to some extent, affected by environmental factors such as nutrition, drugs and stress, autism is not only the sole product of congenital genetic alterations, but may also be elicited by environmental variables via epigenetic mechanisms (Miyake et al., 2012). An example of a hazardous environmental factor that has been linked to ASD (Costa e Silva, 2008) is pesticides (Pearson et al., 2016). *In vitro* studies have found that some pesticides (i.e., rotenone) as well as certain fungicides (i.e., trifloxystrobin, famoxadone, and pyraclostrobin) induce transcriptional modifications that are comparable to those found in brain samples from autistics. Other studies (Kaur et al., 2014; Chauhan and Chauhan, 2015) found that genetically linked mitochondrial dysfunction, associated to increased oxidative stress, (due for example to the exposure to bisphenol A-BPA) is a risk factor for ASD. These findings underscore the importance of uncovering G×E interactions that modulate the emergence of developmental disabilities, even amongst disorders which have been shown to be driven strongly by genetic factors, such as ASD.

#### Exemplar Two: Attention Deficit Hyperactivity Disorder

Besides the studies on ASD (Buchmayer et al., 2009; Maramara et al., 2014), investigations have examined G×E factors behind the etio-pathogenesis of ADHD (Thapar et al., 2012). Potential risk factors for autism span from genes, pre- and perinatal risks to psychosocial variables and environmental risk factors such as toxins (Sciberras et al., 2017; Kalkbrenner et al., 2014). As with ASD, it does not appear that there is a single risk factor underlying the etio-pathogenesis of ADHD. Both genetic and environmental factors interdependently contribute to the disorder, and genes implicated in ADHD overlap with other neurodevelopmental problems, notably, ASD (Lichtenstein et al., 2010; Rommelse et al., 2010; Lundström et al., 2011; Hartman et al., 2016). Several genetic factors, such as having a biological relative with ADHD or possessing minor allele variants have been associated with ASD. Environmental factors, including exposure to lead either pre- or postnatally, severe early childhood adversity, and lower than average birth weight, have been consistently related to autism risk, although none have been proven to be definitely causal. Perhaps unsurprisingly, there is also a large literature documenting association between ADHD and a diversity of putative environmental risk factors that can only be considered as correlates at present (Tarver et al., 2014).

Research paradigms that extend past mere assessments of statistical association have started to contest the robustness of some genetic components which have been previously regarded as ADHD risk factors. Generally, the genetic risks underlying ADHD, while rare, tend to possess small effect sizes and often increase the probability of other psychopathological conditions (Neale et al., 2010; Thapar et al., 2013). Importantly, genetic and environmental factors that modulate the onset of a particular disorder are not necessarily the same ones that shape its course and eventual outcome (Thapar et al., 2007), which, once again, highlights the significance of considering temporal dynamics when pursuing research on developmental disabilities. The influence of genes and the environment do not remain stagnant over the course of development and we have yet to elucidate how G×E interactions evolve over time to contribute to various developmental disabilities.

In sum, although research regarding G×E in developmental disorders has advanced our knowledge base, it has also uncovered a vast expanse of uncharted territory and opportunities for future research inquiries.

### Pathological Self-Generated Environment

Recent articles have shown another potential way through which genetic and environmental factors interact and shape the manifestation of a specific pathological phenotype. Indeed, while environmental hazards represent environmental factors outside of the organism’s control, it is possible for the organism to engage in specific behaviors that modify the environment. These are referred to as pathological self-generated environments, and can create, increase, or reduce the negative impact of the environment on the behavior.

Broadly speaking, each individual reacts differently to the same environment. This can come into play when determining consequences in the environment will trigger distinct responses in the individual. More specifically, this means that the same “starting environment” may trigger diverse outcomes (i.e., “precipitating environment”) on different individuals according to their own responses to the environment. The active generation of environments unique to the individual is based, in part, on one’s genetic propensities, which is a concept known as “active/selective G×E” (Jaffee and Price, 2008). For example, extroverted individuals pursue more socially stimulating environments as compared to individuals who are more withdrawn (Plomin and DeFries, 1979). Consequently, walking into a new office for the first time and saying hello to everyone will most likely generate a different work environment from one in which you walk in and quietly sit down without making eye contact with a soul. This is even truer in the case of pathological self-generated environments, where the pathological characteristics affect the environment in a deeper and potentially more lasting way.

Although at first glance the concept of self-generated environment may seem trivial, in truth, it highlights the strength of the interplay between genetic predispositions and environmental factors, since each interaction between genes and environmental factors precipitates a chain of events which will affect, either deeply or superficially, the subsequent G×E interactions. Therefore, taking into account self-generated characteristics in pathological disorders allows us to acquire a greater understanding of the etiological mechanisms of pathological behaviors and negative environmental situations, ultimately leading us toward the comprehension of the primary cause of the disorder.

However, since this interplay is astoundingly complex, there is a multitude of factors to consider and defining them may not be easy or straightforward. Research into pathological self-generated environments should clearly differentiate the “starting environment” from the “precipitating environment.” Since these environments occur at different instances, it is also critical that researchers consider confounding factors, other than the variables of interest, that may change over time. Assessing a wide range of variables over time will undoubtedly be challenging. To enhance the feasibility of research paradigms investigating self-generated environments, researchers should first identify or shortlist the critical variables involved, either from existing literature or preliminary studies, before directing their efforts on obtaining detailed data on these aspects. Careful selection of experimental designs and operationalization of variables should be conducted prior to these empirical studies.

#### Exemplar One: Autism Spectrum Disorder

**FIGURE 1:**
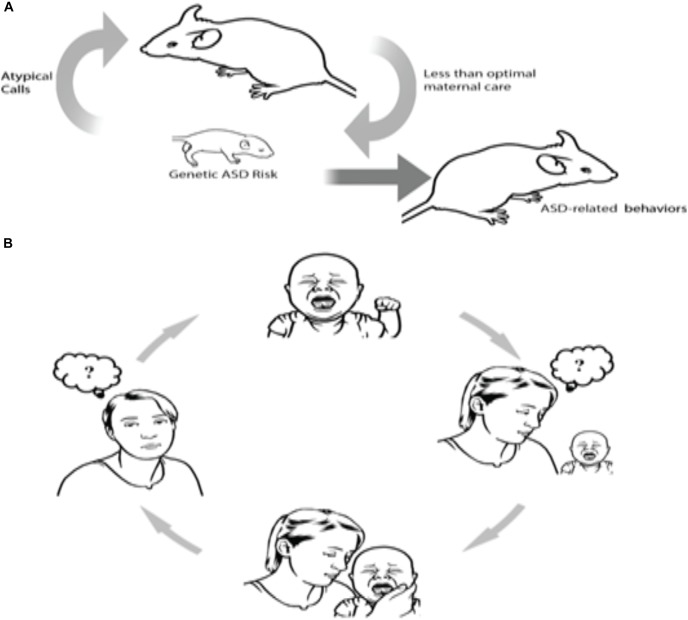
A hypothetical causal chain of events involving atypical vocalizations in mice and humans. Human and mouse data suggest that some genes may lead to the production of atypical distress vocalizations, which in turn, jeopardize effective neonatal social communication between infants and mothers, which in turn worsens outcomes for infants. As illustrated in both rodents **(A)** and humans **(B)**, the cycle proceeds as follows: Atypical vocalization among infants with an ASD genetic risk factor evoke less maternal sensitivity, which in turn can lead to a vicious cycle of confusion and negative perception as to how to appropriately respond to baby’s atypical cries (from Esposito et al., 2017a).

One such example has been recently shown in human and animal models of ASD. Clinically, ASD parents often state that interpreting their autistic infant’s emotional signals is challenging, particularly during the infant’s first year of life. Specifically, they report difficulties in perceiving the reasons for their infants’ distress communications (Esposito and Venuti, 2010; Esposito et al., 2011, 2012; Bornstein et al., 2016). Deficits in understanding the causes behind their infant’s distress vocalizations can trigger a vicious cycle – the mother is unable to recognize and sensitively respond to the infant’s needs, which leads to inadequate feedback that is otherwise required to pacify the infant (Esposito et al., 2011, 2015); this lack of feedback for the infant could then lead to or further compound the child’s regulatory difficulties. In other words, as a consequence of a genetically driven physiological deficit, which compromises the ability of the child to produce typical distress communication signals, caregivers are unable to understand the child’s request and cannot provide adequate sensitive parenting responses to the child (see Figure 1 extracted from Esposito et al., 2017a).

This finding has also been shown in a mouse model of ASD. Indeed, in mice, pups’ vocalizations serve as distress cues for their mothers. Pups’ calls alone are demonstrated to elicit maternal approach behaviors (Uematsu et al., 2007). Encoded in the 22q11.2 region (Hiramoto et al., 2011; Hiroi et al., 2013), *Tbx1* is a monogenic ASD risk gene (Paylor et al., 2006). Takahashi et al. (2015) demonstrated that ultrasound vocalizations (USVs) of pups with a heterozygous deletion of the gene *Tbx1* elicit diminished maternal approach as compared to the USVs of wild-type pups. Contrasted with wild-type littermate pups, the USVs of *Tbx1^−^/Tbx1^−^* pups were also characterized by simpler call sequences and less complex call types. Upon randomized presentation of calls from wild-type and *Tbx1^−^* heterozygous pups, USVs from the former prompted less maternal approach behaviors. This suggests that a mutation of this ASD risk gene modifies neonatal vocalizations, which then renders the pup’s social communication with their mothers ineffective, thus inducing less efficient maternal care (Takahashi et al., 2015; see also Figure 1 extracted from Esposito et al., 2017a). While these findings present convincing evidence for a pathological self-generated environment in autism, another possible mechanism which may govern this phenomenon is pleiotropy, wherein a single gene manipulates the phenotypic expression of a number of disparate traits. In this case, a latent mechanism may stem from an inheritance of a single gene that controls for both reduced maternal sensitivity and atypical vocalizations; this hypothesis has yet to be empirically tested and remains a possible uncontested explanation.

#### Exemplar Two: Internalizing Symptoms

Another example derives from the literature on children’s internalizing symptoms and children’s interpersonal behaviors. Youth with more severe depressive symptoms, related to the expression of 5-HTT, are more likely to seek out for negative feedback and excessive reassurance, behaviors which are thought to serve a cognitive or emotional regulatory function (Caspi et al., 2002). For instance, youth who engage in negative feedback seeking, or the purposeful solicitation of confirmation of negative evaluations from others, are thought to gain a sense of cognitive consistency, which may enhance feelings of control. Ironically, however, both of these interpersonal behaviors are prospectively associated with worsening social relationships, in the form of exacerbating perceptions of friends’ criticism and decreased friendship quality. In addition, these interpersonal behaviors are associated with increased depressive symptoms over time (Prinstein et al., 2005; Borelli and Prinstein, 2006). Thus, youth at risk for higher depressive symptoms, such as carriers of the minor serotonin transporter allele, may have more vulnerable self-concepts and behave in ways that elicit from others the interpersonal reactions they fear – this self-fulfilling prophecy provides another instance of environmental effects that relate, at least in part, to genetic characteristics.

With only a handful of examples, we hope to have illustrated what we view as a central consideration within the field of studying G×E interactions – the fact that many of the environmental factors we examine are in fact partly dependent upon genetic factors. Thus, studying true G×E interactions becomes infinitely more challenging than examining statistical interactions between genes and environment.

## Challenge Four: Choosing a Developmental Period

The interaction of G×E plays a major role in determining developmental trajectories. As such, the timing of the measurement of individual difference factors is of key import.

### Exemplar: G×E Informing Understanding of the *Development* of Psychopathology

Perhaps the most well-known example of how the study of G×E has impacted our understanding of developmental psychopathology hails from Caspi et al. (2003). These researchers showed that functional polymorphisms of a specific gene (MAOA) may modulate the likelihood of developing antisocial tendencies. Not only do these findings provide epidemiological evidence that support how genetic factors modulate children’s sensitivity to environmental insults, they may also account for differences in developmental trajectories, and why not all victims of maltreatment victimize others later in life. In another study from the same group, Caspi et al. (2002) have shown how the impact of stressful life events (SLEs) on depression was moderated by a functional polymorphism in the promoter region of the serotonin transporter (5-HTT) gene. As compared to homozygotes who have two copies of the long allele, persons in possession of at least one copy of the short allele of the 5-HTT promoter polymorphism displayed higher levels of depressive symptoms and suicidality, and are more likely to be diagnosed with depression. In another study related to 5-HTT (Kochanska et al., 2011), researchers documented that an effect of developmental trajectories on children’s social and cognitive development was due to interactions between children’s serotonin transporter linked promoter region [5-HTTLPR] gene and maternal responsive care, observed at different time points during development (15, 25, 38, and 52 months). Specifically, children’s genetic make-up moderated the impact of responsive maternal care on every domain of children’s competence. Among children who have at least one short 5-HTTLPR allele (s/s, s/l), those with more responsive mothers were significantly more adept as compared to those with less responsive mothers. Interestingly, responsiveness had no discernible effect on children with two long alleles (l/l). The interaction of specific gene polymorphisms with early environmental factors (i.e., early caregiving experiences) has been reported in several other studies, suggesting how it plays a major role in differential developmental trajectories of responses to social stimuli (Esposito et al., 2016; Senese et al., 2016), conduct disorder (Brody et al., 2011), externalizing behavior (Brett et al., 2015), emotion regulation and depressive symptoms (Borelli et al., 2016).

Given the extensiveness of the human genome, it is only reasonable that another level of complexity, gene × gene (G×G) interaction, is at work as well. Some of the same genes (i.e., MAOA and COMT) involved in G×E interactions have been implicated in G×G studies. In a study on OCD, McGregor et al. (2016) revealed a G×G interaction of rs362204 (COMT) and rs1799836 (MAOB), and rs362204 (COMT) and rs6651806 (MAOB) in contributing to the pathology of OCD. Additionally, a G×E interaction of childhood severity with variants of COMT, MAOA, and MAOB was also found in relation to OCD. While G×G and G×E paradigms capture genetic and environmental contributions, a more intricate G×G×E study has recently been attempted by Wang et al. (2018). Indeed, they discovered a 3-way interaction of MAOA, COMT and SLEs on adolescents’ aggressive behavior, demonstrating the complicated interplay of genetic and environmental interactions in shaping development.

### Epigenetic Effects

Great interest has recently been given to studies on epigenetics, specifically, on DNA methylation. Epigenome-wide association studies (EWAS) is a technique that has been developed to further investigate G×E interactions. EWAS computes DNA methylation markers on the premise that epigenetic methylation is a phenomenon influenced by both extrinsic environmental factors and the genetic code (Ng et al., 2012; Murphy and Mill, 2014; Holbrook, 2015). Within the same chromosome (*cis*-acting), SNPs which are proximal to CpGs tend to produce methylation quantitative trait loci (methQTLs) (Gibbs et al., 2010; Zhang et al., 2010; Bell et al., 2011), which can be quantified using software suites such as the Gene, Environment, Methylation (GEM) toolkit (Pan et al., 2016). One startlingly comprehensive example is the methylation of FKBP5, which, among individuals with a risk allele, is reduced upon exposure to childhood trauma. The extent of methylation has also been evidenced to predict risk of psychopathology in adulthood (Klengel and Binder, 2013, 2015). Using methylation as a proxy of G×E interaction, Teh et al. (2014) showed that variations in the infant’s methylation profile is most suitably explained by an interaction between the prenatal environment and a specific SNP.

Although assessment of methylation levels is still under debate, it has been suggested that saliva-derived samples for methylation analysis might present the field with tremendous opportunities for non-invasive epigenetic studies on typical and atypical developmental trajectories (van IJzendoorn et al., 2011). An example in this field is offered by the study of Schechter et al. (2016), which reported an association between serotonin receptor 3A methylation with maternal violence exposure, with the development of children’s neural activity and antisocial behavior.

### Response to Interventions

It is also within this domain of research on developmental trajectories that the onus for the creation of interventions lies. Identifying how gene–environment interaction modulates the mechanisms of disease development is paramount to identifying patients with an inherent vulnerability to certain conditions. This identification in turn may allow patients to be targeted with individualized treatment based on the knowledge of their inborn susceptibility to specific conditions (Harding, 2007). Certain developmental disabilities, such as ADHD (Huss et al., 2017) and intellectual disabilities (Tsiouris, 2010; Paton et al., 2011; McQuire et al., 2015), have been shown to benefit from pharmacological interventions. In the field of developmental disabilities, pharmacological treatments require development using animal models. However, not all symptoms of disorders, critical in the diagnosis of psychopathology in humans, is measurable in animal models. As a result, animal models of these disorders are as assorted as the disorders themselves, reconstructing some but not entire features of the disorder (Cope et al., 2016). This non-standardization of what constitutes a recreation of a specific disorder in an animal may partially explain why translational treatments derived from these models have yet to successfully cure the targeted patient populations. Besides the problem of transmuting findings from animal models to humans, further hurdles stem from inconsistencies in the environmental factors used in these models that may contribute to a myriad disorders. Moreover, these factors may influence specific psychological domains, cutting across various disorders. This limits translational efficacy since individual disorders are currently defined by diagnostic boundaries.

The next revolution in psychopathological intervention is geared toward tailoring treatment approaches to individuals based on one’s characteristics and specific needs (Belsky and van Ijzendoorn, 2015). Taking a step further in this challenging direction, proponents of experimental-intervention research also assert the importance of considering differential genetic vulnerability to environmental stressors (e.g., Beach et al., 2010; Kegel et al., 2011), and others have made observations that support this (Belsky et al., 2007; Belsky and Pluess, 2009, 2013; Ellis et al., 2011). Knowing which individual traits modulate the impact of treatment allows therapists to adjust treatment programs accordingly so as to deliver maximum benefits. Without taking differences in susceptibility into account, treatment effects would appear larger for those who carry a greater genetic predisposition, and smaller for those who are less predisposed (Belsky and Pluess, 2009, 2013; van IJzendoorn et al., 2011). As a result, treatment might be misconstrued as ineffective, when in actuality, the success of intervention programs is influenced by the genetic basis of differential susceptibility in individuals. A paradigm shift that aims to understand the manner in which genetic variables interact with the environment will shed greater light on why treatment is rendered differentially efficacious (Meaney, 2010; van IJzendoorn et al., 2011). While ethical concerns such as stigma and discrimination must be addressed (Ellis et al., 2011), knowledge of individuals’ genetic predisposition undoubtedly serves as invaluable information that informs therapists on how to best structure therapy intervention to further alleviate psychopathological distress.

The genes of serotonin transporter linked promoter region (5-HTTLPR) and dopamine receptor D4 (DRD4) have shown to have an impact on infant temperament and attachment style and to modulate the intervention efficacy (Cicchetti et al., 2011; Holmboe et al., 2011). In another study on intervention in depressive symptoms (Cicchetti et al., 2015), changes in symptomatology over time depended on genotypes (5-HTTLPR and CRHR1) and the type of psychological intervention they were receiving. Other studies focusing on dopamine D4 receptor polymorphism have found a G×E interaction in modulating intervention effects in literacy-delayed children (Plak et al., 2015), in toddlers’ externalizing behavior (Bakermans-Kranenburg et al., 2008), and in preventive intervention on adolescent drug abuse (Brody et al., 2014, 2015).

Response to psychotherapy has also been related to level of DNA methylation. For instance, among individuals with borderline personality disorder, outcome of psychotherapy was related to methylation status of the BDNF gene, with the findings suggesting that, over time, variations in methylation status were considerably linked to changes in depression, hopelessness, and impulsivity scores (Perroud et al., 2013). In another study, responses of children with anxiety disorders to cognitive behavior therapy was associated with an increase in serotonin transporter methylation (Roberts et al., 2014).

## G×E Challenges

Although the number of G×E studies is rising with astounding alacrity, as with any other field, research in this area bears an indubitable set of challenges and limitations (Dick et al., 2015). In this section, we address the main challenges that beset this field and present suggestions on how to overcome them.

An issue that is of foremost concern from a methodological standpoint is the widespread implementation of candidate-gene studies. In such approaches, the mechanistic function of specific genes known to be linked to the psychiatric disorder of interest forms the basis of selecting them as genetic factors. Subsequently, researchers will investigate if there exists an interaction between variants in this gene (e.g., polymorphisms), and certain environmental conditions, in predicting the likelihood of the development of the psychiatric disorder. Despite numerous publications that have emerged from candidate-gene approaches, findings from these studies are often not consistently reported across multiple experiments. This lack of reproducibility seems to stem from the small sample sizes that often characterize candidate-gene studies (<1,000 participants), which consequently leads to low statistical power in detecting G×E interactions with small effect sizes. Since studies with larger sample sizes result in greater statistical power that can make this distinction, one might think that a straight-forward solution to this problem lies in advocating for larger sample sizes. However, in doing this, an unfortunate trade-off is the loss of a rich plethora of environmental information that could have otherwise been acquired in smaller sample sizes. To illustrate this point, consider a study that aims to investigate how specific genetic variants interact with the family environment to influence the development of psychiatric disorders: Should the study collect detailed environmental data (e.g., through numerous sessions of behavioral observations in a naturalistic home setting)? To answer this, we must consider that the likelihood of obtaining a massive number of participants in the thousands is small as only a handful of families would be agreeable to such arrangements and the research staffing necessary to complete this type of investigation is large. Conversely, a less complex method of obtaining data of the environment (e.g., by having a parent report on family environment using a self-report measure) may be an alternative solution, albeit generating less nuanced information would be more feasible and palatable to families. Researchers are hence faced with the difficulty of balancing these trade-offs of quality for quantity. A possible way of circumventing this obstacle is to increase collaborations across research groups, such that samples from numerous candidate-gene studies that are conducted in different locations are pooled to form a larger sample size. Another possible approach is to identify methods of collecting environmental data that is both convenient (i.e., short duration) and allows for retrieval of a rich amount of environmental information.

The discussion above primes us for the next issue to be addressed, which pertains to the lack of systematic examination of environmental variables in G×E studies. Despite an extensive psychiatric literature, it is uncommon for studies to consider both the positive and negative ends of the environmental spectrum (Bakermans-Kranenburg and van Ijzendoorn, 2006; Taylor et al., 2006), which leads to a restricted range of environments being investigated and an incomplete depiction of the development of psychiatric conditions (Belsky and Pluess, 2009). For example, studies which are interested in the effects of negative life events would categorize the absence of environmental stressors on the extreme end of the negative scale (e.g., Caspi et al., 2003), thus failing to consider the positive portion of the spectrum altogether. As a result, the nature of the G×E interaction under investigation can be incorrectly understood, with a risk of possibly identifying the presence of a G×E interaction when in fact, there is none. This leads to spurious associations of genetic and environmental variables and gives rise to high false discovery rates (Duncan and Keller, 2011). Researchers such as Patel et al. (2013), who have begun to address this gap in the literature, recommend generating high-throughput environmental data to distinguish key elements in the environment that contribute to shaping of psychiatric disorders. Broadening the scope and accuracy of critical environmental data being analyzed should ideally be accompanied by incorporation of a “differential susceptibility” and “plasticity” framework, such that positive and negative environmental influences are addressed in G×E studies at both the theoretical and methodological levels.

Since G×E research lies directly in the intersection of psychology and genetics, it is not surprising that increasing attention on this topic has been received from geneticists (Engelman et al., 2009). Despite ever increasing collaborations between these two fields, fundamental differences in the theoretical and practical approaches toward G×E studies continue to persist (Risch et al., 2009; Caspi et al., 2010). The challenges that have surfaced further cement the need for G×E studies to be validated beyond these specialties alone, with cross-disciplinary assessments ideally being replicated on a mechanistic level in the areas of neurobiology, neuroimaging, and other related disciplines. Indeed, to reduce high false discovery rates, Rutter et al. (2006) propose that hypotheses for these studies should stem from an understanding of potential biological pathways that integrate genetic and environmental influences, rather than searching for an interaction from open-ended statistical manipulations. For instance, since the discovery that individuals with two copies of the S-allele of 5-HTTLPR, who have been exposed to adverse environments, are at greater risk of suicide (Caspi et al., 2003), subsequent studies revealed that 5-HTTLPR also interacts with various environmental stressors to moderate the onset of a broad span of psychiatric disorders, such as depression, anxiety, ADHD, and eating disorders (Gibb et al., 2006; Kranzler et al., 2012; Stoltenberg et al., 2012; van der Meer et al., 2014; Liu et al., 2015). Likewise, in accordance with the differential susceptibility hypothesis, homozygotes of the S-allele who were exposed to supportive environments exhibited the fewest depressive symptoms (Eley et al., 2004; Taylor et al., 2006). Neuroimaging studies have also revealed promising findings that converge with those emerging from these G×E studies. Specifically, distinct patterns of activation in areas of the brain (e.g., amygdala) that are implicated in emotional processing have been observed for different genotypes of 5-HTTLPR (Canli et al., 2006; Munafò et al., 2008; Fortier et al., 2010; Alexander et al., 2012). Alteration in emotional processing based on different genotypes also manifest at the level of the autonomic nervous system. Congruent with brain imaging analyses, Mueller et al. (2012) reported that children who are carriers of the L-allele, as compared to those who are S-carriers, exhibited a greater increase in salivary α-amylase, which is suggestive of a faster recovery after exposure to stressors in individuals who are carriers of the L-allele. Each of these multi-level findings contribute a piece of information that stitches the pathway through which 5-HTTLPR exerts its effects. Findings from various fields point to a possible biological mechanism: differences in alleles of the serotonergic receptor dictates differences in reactivity of the brain toward stressors, and subsequently increases the risk of a dysregulated emotional processing and stress response system in S-allele carriers.

In the long-run, a comprehensive understanding of the biological mechanisms through which genetic differences lead to the development of psychiatric disorders allow for numerous possibilities in customization of individualized treatment. Individualized intervention aims to accurately profile a person’s psychiatric diagnosis and verify the most effective mode of treatment (Ozomaro et al., 2013). In an effort to enhance treatment efficacy, extensive research into biological markers (Garriock et al., 2006; Ising et al., 2009; Keers and Aitchison, 2011) and sociodemographic indicators (Nanni et al., 2012; Nemeroff et al., 2003) have been utilized to predict treatment response but both approaches have elicited inconsistent findings. These findings suggest that neither genetic variants, nor environmental variables, can predict an accurate prognosis, whereas a consideration of the combination of both factors (i.e., G×E interaction) might possibly lead to more precise predictions that will be of invaluable assistance in treating psychiatric disorders.

## Conclusion

Our hope is that this special issue raises interesting research questions and ideas that can be further explored in future investigations. We contend that the following goals are essential to pursue in the next decade of research. First, we are direly in need of more studies on G×E in developmental disabilities. Second, we must refine and articulate a clearer understanding of environmental hazards as opposed to self-generated dynamics, with the latter being more amenable targets of prevention and intervention efforts.

In sum, a multitude of developmental disorders emerge from the encounter of a generic genetic susceptibility and a specific environmental context (Kovas and Plomin, 2007). After the identification of this interaction, the first step to develop efficient treatments requires development using animal models (Cope et al., 2016). However, animal models of developmental disorders are as assorted as the conditions themselves, often unable to mimic all aspects of the disorder. For this limitation to be surmounted, greater accuracy in the replication of disorders in animal models needs to be obtained. A possible means of achieving this is through recognizing common endophenotypes that exist across both animal and human populations. The next frontier may be the stratification of developmental disorders based on both genetic and environmental markers. This would help to decrease the heterogeneity of the targeted population and will possibly identify specific endophenotypes that may be targeted in animal models, enhancing translational efficacy.

## Author Contributions

GE, AA, and JB have conceived, designed, and wrote the paper.

## Conflict of Interest Statement

The authors declare that the research was conducted in the absence of any commercial or financial relationships that could be construed as a potential conflict of interest.

## Footnotes

1 https://www.nimh.nih.gov/research-priorities/rdoc/nimh-rdoc-publications.shtml
